# The impact of religiosity, anxiety and depression on proneness to auditory hallucinations in healthy individuals

**DOI:** 10.1192/bjo.2025.10775

**Published:** 2025-07-22

**Authors:** Chiara Lucafò, Irene Ceccato, Gianluca Malatesta, Rocco Palumbo, Nicola Mammarella, Alberto Di Domenico, Luca Tommasi, Giulia Prete

**Affiliations:** Department of Psychology, University of Chieti-Pescara, Chieti, Italy

**Keywords:** Auditory hallucinations, voice hearing, religiosity, depression, anxiety

## Abstract

**Background:**

Auditory hallucinations (hearing voices in the absence of physical stimuli) are present in clinical conditions, but they are also experienced less frequently by healthy individuals. In the non-clinical population, auditory hallucinations are described more often as positive and not intrusive; indeed, they have received less attention.

**Aims:**

The present study explores the phenomenology of non-clinical auditory hallucinations and their possible relationship with religiosity.

**Method:**

Starting from previous findings suggesting that non-clinical auditory hallucinations are often described as a gift or a way to be connected with ‘someone else’, we administered standardised questionnaires to quantify proneness to experiencing auditory hallucinations, religiosity and anxiety/depression scores.

**Results:**

Regression analysis carried out using an auditory hallucinations, index as the dependent variable on a final sample of 680 responders revealed that a total of 31% of the variance was explained by a five-steps model including demographic characteristics (i.e. being young, a woman and a non-believer) and negative (e.g. being afraid of otherworldly punishments) and positive (e.g. believing in benevolent supernatural forces) components of religiosity, anxiety and depression. Crucially, compared with believers, non-believers revealed higher scores in depression, anxiety and in a specific questionnaire measuring proneness to auditory hallucinations.

**Conclusions:**

Results suggests that religiosity acts as a potential protective factor for proneness to paranormal experiences, but a complex relationship emerges between religious beliefs, mood alterations and unusual experiences.

## Clinical and non-clinical auditory hallucinations

A significant proportion of healthy individuals hear voices that others cannot hear.^1^ Hearing voices, or auditory hallucinations, can be defined as a perception of auditory inputs in the absence of corresponding physical stimuli, and their manifestation lies on a continuum from pathology to normality.^2^ auditory hallucinations are relatively common in several psychotic disorders, such as schizophrenia and dissociative identity disorder:^3^ auditory hallucinations are present in about 70% of schizophrenic patients and 11–63% of patients diagnosed with bipolar disorder and depression.^4^ From a neurophysiological point of view, clinical auditory hallucinations (i.e. psychiatric symptoms) are related to abnormal activity of linguistic areas in the left hemisphere and to reduced communication activity between the two sides of the brain. From a phenomenological point of view, they are often described as male voices, conveying negative content in an intrusive and uncontrolled manner.^5^

It is less known, however, that auditory hallucinations are also relatively frequent in the non-clinical population, with 4–15% of the healthy adult population hearing voices.^6^ In fact, hearing voices can also be considered a common human experience and a normal mental process:^7^ non-clinical auditory hallucinations are a still debated issue, and a very recent, growing literature concerns the possible role of religiosity and/or spirituality in healthy persons experiencing auditory hallucinations,^8^ even if this topic has been scarcely explored and needs more attention.

## Spirituality and auditory hallucinations

The voices heard are often referred to as a higher self or a supernatural entity – for example, angels, spirits, demons.^9^ According to some studies, between 10 and 25% of people have experienced auditory hallucinations at least once in their lives,^10^ and in some cultures hearing voices is considered to be a religious experience.^11^ However, individuals who perceive voices as a manifestation of mental illness (i.e. clinical auditory hallucinations) tend to perceive them as distressing, whereas those who perceive voices as a manifestation of spiritual experience (i.e. non-clinical auditory hallucinations) tend to perceive them with greater positivity, almost like ‘a gift’.^11^ Moreover, within the subset of individuals reporting experiences of auditory hallucinations, higher religious belief seems to be correlated with increased pleasantness,^12^ reduced distress and enhanced perceived mastery of these unusual experiences.^13^ According to Cottam and colleagues,^12^ religious belief appears to exert a significant and advantageous influence on mentally healthy Christians’ perception of hearing voices, who perceive voices as powerful, positive and enabling them to fulfil God’s will and to enhance resilience towards adverse events in daily life.^14^ Moreover, it has been found that 58% of healthy participants with auditory hallucinations believed their voices came from ‘benevolent spirits’,^15^ and a study revealed that 91% of healthy individuals with auditory hallucinations defined themselves as ‘spiritualist’, compared to 41% of non-voice-hearing controls and 76.5% of psychosis patients.^16^ An online survey in a sample of 58 predominantly Christian participants^17^ showed that almost 90% of the sample reported that voices were divine in identity and approximately 30% were heard in the context of prayer, and these voices were described as including personal significance, positive emotions and occurring in the context of praying.

## Phenomenology of clinical and non-clinical auditory hallucinations

Daalman and colleagues^5^ compared 101 psychotic patients with 101 non-clinical voice-hearers, and found that auditory hallucinations in the non-clinical population are less frequent and more controllable, with the negative emotional valence of the content of auditory hallucinations predicting the presence of a psychotic disorder in 88% of the sample (see also^18^). From a phenomenological viewpoint, a survey on the spatial source of voices carried out on 198 psychiatric patients^19^ revealed that 47% of patients heard ‘internal’ voices and38% heard ‘external’ voices, without significant difference between the left and the right side. Coming back to non-clinical auditory hallucinations, it has been shown that when healthy participants are asked to imagine hearing a voice in one ear, they significantly reported it more frequently in the right ear than in the left^20^ (see also^21^), and this asymmetry has been ascribed to the left-hemispheric superiority for language, which would be altered in patients suffering from auditory hallucinations. Moreover, when emotional valence is called into question, it has been shown that this right-ear advantage is stronger for voices expressing positive contents,^22^ indirectly confirming hemispheric asymmetry during voice-hearing/imagery as a ‘protective’ factor against the negative valence of clinical auditory hallucinations. Importantly, the same imagery protocol did not show asymmetry in patients suffering from clinical auditory hallucinations.^23^

The recent literature concerning the difference between clinical and non-clinical auditory hallucinations opens a question about the possible link between spirituality and the ‘ability’ to hear voices, drastically changing the attribution of auditory hallucinations, from a psychiatric symptom (clinical auditory hallucinations) to a ‘special ability’ (non-clinical auditory hallucinations). It is useful here to provide a definition of ‘spirituality’, as the belief in something intangible, including – but not limited to – religiosity (divinity, resurrection or reincarnation), spirit possession, interaction with spirits, supernatural events, superstition and connection with the dead.^24^ Of note, an association between, for instance, religiosity and an enhanced prevalence of psychotic symptoms has been already proven, explained as due both to exploiting religion as a coping strategy (consequence) for psychotic symptoms and to religion as a tool (reason) of belief in supernatural forces and thus supernatural perceptions.^25^ Besides this kind of literature, which is mainly focused on visual hallucinations, recent studies add important hints in the auditory domain, showing that in spiritualist mediums voice-hearing precedes spiritual beliefs^26^ and confirming that the content of such voices is positive and elicits a positive feeling.^27^

## Possible role of depression and anxiety in auditory hallucinations

Several authors have proposed an important role for anxiety and depression in the formation and maintenance of hallucinations and persecutory delusions: research suggests that depressive symptoms tend to be more frequently associated with delusional beliefs and hallucinatory experiences across both non-clinical and clinical populations,^28^ while anxiety symptoms tend to be more frequently associated with hallucinatory episodes in the general population.^29^ Smith and colleagues^33^ found an association between higher levels of depression, lower self-esteem and the strength of auditory hallucinations and persecutory delusions, thus suggesting a linear relationship between positive symptoms and depression; and it has been found that anxiety levels significantly increased as the frequency of auditory hallucinations increased.^31^ These findings underscore fundamental connections between anxiety, depression and the manifestation of delusional beliefs and hallucinatory phenomena.

## The present study

Starting from this complex frame, the present study aimed to shed light on the frequency and phenomenology of hearing voices in the non-clinical population. A second aim was exploring the possible relationship between hearing voices and supernatural beliefs (e.g. religiosity level). By means of an online survey, we collected data from a large sample and exploited statistical models to explore the links between hearing voices, possible laterality biases, religiosity, anxiety, depression and demographic features in a sample of individuals without a psychiatric diagnosis. We expected the level of measured depression and anxiety to partially explain the frequency and intensity of auditory hallucinations in the non-clinical population, and we wondered whether religiosity affects this frame, possibly contributing to create a protective filter in persons experiencing auditory hallucinations compared to persons with less spiritual beliefs.

## Method

### Participants

A total of 958 responders took part in the study. The sample size was not specifically calculated *a priori* (also due to the lack of previous similar available data), and we decided to keep the link active until at least 600 responders fully completed the survey. From the initial sample, persons who declared neurological/psychiatric conditions and/or did not complete the survey were excluded. Due to the scarcity of participants over 40 years of age (*N* = 15) and with a level of education lower than high school diploma (*N* = 2), these participants were also excluded from the analyses to obtain a sample as homogeneous as possible. The final sample constituted 680 healthy Italian participants (493 females and 187 males), including 335 self-declared non-believers and 345 believers (derived from a dichotomous item about general religiosity: ‘Do you have a religious belief? If yes, what religion do you believe in?’). The age of the sample ranged from 18 to 40 years (mean 20.54; s.e. ± 0.13). The link was distributed among university students who were also asked to share it with friends and parents. This recruitment strategy could explain why approximately 95% of respondents were students, potentially introducing a sample bias. Demographic data in frequencies are reported in Table 1.

**Table 1:** Demographic information of the final sample (and proportion of the whole sample: *N* = 680)

Sex	Female	Male		
	493 (0.725)	187 (0.275)		
Education	High school	Bachelor’s degree	Master’s degree	Post-graduate degree
	643 (0.945)	21 (0.031)	11 (0.016)	5 (0.008)
Work	Student (unemployed)	Unemployed	Employee	Private worker
	614 (0.903)	6 (0.009)	45 (0.066)	15 (0.022)
Marital status	No answer	Single	Engaged	Married
	22 (0.032)	353 (0.519)	299 (0.44)	6 (0.009)
Being a believer	Non-believer	Believer		
	335 (0.493)	345 (0.507)		
Religion	Christianity	Buddhism	Islam	Other
	326 (0.479)	3 (0.004)	3 (0.004)	13 (0.019)
Handedness	Left-handers	Right-handers		
	63 (0.093)	617 (0.907)		

### Procedure

To explore the phenomenology of non-clinical auditory hallucinations, including their relationship with religiosity, an online survey was created and shared online by using Qualtrics XM. Before starting the survey, participants provided their informed consent on a form that described the study aims, data treatment procedure and participant rights. The entire procedure took approximately 30 minutes to complete, and at the beginning of the survey participants were informed that all data would be treated confidentially on their signing the informed consent to participation document online. All procedures contributing to this work comply with the ethical standards of the relevant national and institutional committees on human experimentation and with the Helsinki Declaration of 1975, as revised in 2013. All procedures were approved by the Institutional Review Board of Psychology – Department of Psychological, Health and Territorial Sciences, Università degli Studi ‘G. d’Annunzio’ Chieti-Pescara (protocol number: IRBP/22011).

The survey was composed of different sections aimed at collecting sociodemographic information, psychological data and assessing the phenomenology and affective experiences of hearing voices. Specifically, sections included measures of laterality preference (Edinburgh Handedness Inventory: EHI^32^), depression (Beck Depression Inventory: BDI^33^), anxiety (State-Trait Anxiety Inventory: STAI-Y2), proneness to experiencing paranormal events (Cardiff Anomalous Perceptions Scale: CAPS^36^ and Unusual Experience Scale from O-LIFE (Oxford–Liverpool Inventory of Feelings and Experiences)^37^), auditory hallucinations (Auditory Hallucinations Rating Scale: AHRS^38^ and Hamilton Program for Schizophrenia Voices Questionnaire: HPSVQ^39^), frequency of hallucinatory experiences (HE^17^) and religiosity (Positive and Negative Religious Coping: RCOPE^40^). Details of each measure are provided in the descriptive statistics section below. When unavailable, the Italian translation was made *ad hoc* and validated by a bilingual person.

### Statistical analysis

**Fig. 1:**
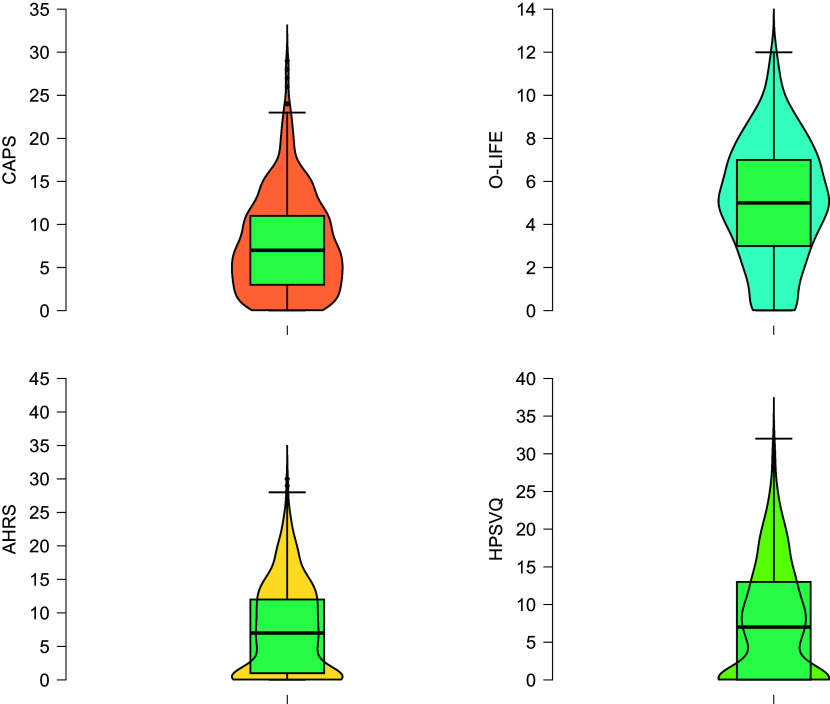
Distribution plots for auditory hallucinations questionnaires. The thick horizontal line indicates the median; the box indicates the interquartile range (IQR); the whiskers indicate extreme data points not exceeding the IQR by1.5; outliers are plotted as dots. CAPS, Cardiff Anomalous Perceptions Scale; O-LIFE, Oxford–Liverpool Inventory of Feelings and Experiences; AHRS, Auditory Hallucinations Rating Scale; HPSVQ, Hamilton Program for Schizophrenia Voices Questionnaire.

As a first analysis, correlations were computed between the questionnaires used to evaluate auditory hallucinations (i.e. CAPS, AHRS, HE, O-LIFE, HPSVQ). Then parallel analysis with 5000 replications (geomin rotation, maximum likelihood estimator) and principal component analysis (PCA) was used to compute the shared variance among these measures for dimensionality reduction. The resulting factor score was saved (regression method) and used in the following analyses as a single index of auditory hallucinations (see Table 2 and Fig. 1 for descriptives and distributions). A hierarchical linear regression was conducted to evaluate the influences of demographic factor, religiosity and psychological variables on the index of auditory hallucinations. Demographic information (age, sex, being or not being a believer and laterality score) was included in the first step. Religiosity scores were entered in two subsequent steps, to elucidate the independent effect of negative religiosity (RCOPE-neg) and positive religiosity (RCOPE-pos) on hallucinations and anomalous perceptual experiences. Psychological variables including anxiety (STAI-Y2) and depression (BDI-II) were added separately in the two final steps.

**Table 2:** Range of responses, mean and s.e. and Cronbach’s α for each questionnaire of the whole sample (*N* = 680)

Questionnaire	Range	Mean (s.e.)	Cronbach’s *α*
Edinburgh Handedness Inventory (EHI)	−100/+100	57.82 (1.58)	0.94
Beck Depression Inventory (BDI)	0/63	11.91 (0.32)	0.876
State-Trait Anxiety Inventory (STAI)	1/80	47.17 (0.42)	0.918
Cardiff Anomalous Perceptions Scale (CAPS)	0/32	2.51 (0.07)	0.864
Unusual Experience Scale (O-LIFE)	0/12	5.02 (0.1)	0.728
Auditory Hallucinations Rating Scale (AHRS)	0/40	7.54 (0.26)	0.825
Hamilton Program for Schizophrenia Voices Questionnaire (HPSVQ)	0/36	7.71 (0.28)	0.934
Hallucinatory experience (HE)	0/4	1.70 (1.53)	n.a.
Positive Religious Coping (RCOPE-pos)	7/28	11.24 (0.19)	0.928
Negative Religious Coping (RCOPE-neg)	7/28	10.12 (0.15)	0.826

## Results

Data were analysed using IBM SPSS 29 and Mplus 7 for Windows.^41^ To illustrate the prevalence of auditory hallucinations in the sample, score ranges, means, standard deviations and Cronbach’s α of all the measures are reported in Table 2 and the distributions of measures assessing auditory hallucinations are depicted in Fig. 1. Descriptive statistics for all measures are reported in the Supplementary material available at https://doi.org/10.1192/bjo.2025.10775.

### Anomalous experiences: auditory hallucinations index

Anomalous perceptions were not uncommon in the general population, as indicated by the CAPS and O-LIFE questionnaires. Only 7% of participants reported no hallucinations/anomalous experiences at all, while the majority of participants reported at least one unusual experience during their lifespan. For instance, 88% of participants felt that their accidents were caused by mysterious forces (O-LIFE, item 10), and more than 90% stated they had the sensation of not being in full possession of their limbs, or that common objects looked abnormal (CAPS, items 10 and item 26). Mean scores for CAPS and O-LIFE were in line with previous studies in non-clinical populations.^36^ Of note, in the questionnaires exclusively focused on auditory hallucinations (HPSVQ, AHRS, and HE) the prevalence of auditory hallucinatory experiences was relatively low. Indeed, 31, 23 and 35% of participants reported scores of zero for HPSVQ, AHRS and HE, respectively.

Correlations between the questionnaires used to evaluate auditory hallucinations (i.e. CAPS, AHRS, HE, O-LIFE, HPSVQ) ranged from *r* = 0.35 to *r* = 0.84, *p* < 0.001. Results from the parallel analyses with 5000 replications (geomin rotation, maximum likelihood estimator) suggested the retention of a single factor. Similarly, the PCA identified a single component explaining 64% of variance. Factor loadings ranged from 0.89 to 0.69, and the resulting factor score was used as a single index of auditory hallucinations in the hierarchical linear regression.

### Linear regression explaining auditory hallucinations index

Results of the hierarchical linear regression indicated that demographic variables entered in the first step collectively explained 11% of the variance in the auditory hallucinations index: *F*(4, 675) = 20.48, *p* < 0.001. Being younger, being a woman and being a non-believer were significantly associated with higher auditory hallucinations (age: *B* = −0.06, s.e. = 0.01, β = −0.20, *t* = −5.59, *p* < 0.001; gender: *B* = 0.53, s.e. = 0.08, β = 0.24, *t* = 6.55, *p* < 0.001; belief: *B* = −0.15, s.e. = 0.07, β = −0.08, *t* = −2.12, *p* = 0.034). Laterality had no impact on auditory hallucinations: *B* = 0.10, s.e. = 0.09, β = 0.04, *t* = 1.14, *p* = 0.257. Entering RCOPE-neg significantly improved explained variance: ΔR^2^ = 0.07, *F*(1, 674) = 58.92, *p* < 0.001. Furthermore, the addition of RCOPE-pos in the subsequent model further improved explained variance: ΔR^2^ = 0.01, *F*(1, 673) = 12.23, *p* < 0.001. This suggests that both negative and positive religiosity predicts hallucinatory experiences. Lastly, both STAI-Y2 and BDI explained significant amounts of additional variance: ΔR^2^ = 0.11, *F*(1, 672) = 101.85, *p* < 0.001 and ΔR^2^ = 0.01, *F*(1, 671) = 7.96, *p* = 0.005, respectively – namely, higher levels of anxiety and depression explain greater auditory hallucinations. Overall, the final model explained 31% of variance in auditory hallucinations. Coefficients of the final step are presented in Table 3. Globally, this pattern of results showed a complex relationship between auditory hallucinations and religiosity, and revealed that other factors (e.g. anxiety) play a major role.

**Table 3:** Regression coefficients for the final model (step 5) predicting the auditory hallucinations index

*Predictor*	*B*	s.e.	*β*	*t*	*p*
Age	−0.05	0.01	−0.16	−4.84	<0.001
Sex	0.30	0.08	0.13	3.93	<0.001
Being a believer	−0.25	0.08	−0.13	−3.21	<0.001
Laterality	0.11	0.08	0.05	1.41	0.159
RCOPE-neg	0.04	0.01	0.16	4.36	<0.001
RCOPE-pos	0.03	0.01	0.13	3.03	0.003
STAI-Y2	0.02	0.01	0.24	4.389	<0.001
BDI-II	0.02	0.01	0.15	2.82	0.005

Sex was coded: male = 0, female = 1. Being a believer was coded: non-believer = 0, believer = 1.

### Impact of religious belief

Starting from the effect of being believer found in the regression analysis, we explored potential differences between believers (*N* = 345) and non-believers (*N* = 335) on different variables. A series of analyses of variance (ANOVA) was carried out using as the between-subjects factor the dichotomous self-assessment of being a believer or not. First, the ANOVA confirmed a higher religiosity level in believers than in non-believers both in the RCOPE-pos (believers: 13.87 ± 5.40; non-believers: 8.53 ± 2.65, *F*(1, 678) = 265.33, *p* < 0.001) and in the RCOPE-neg (believers: 10.81 ± 4.07; non-believers: 9.41 ± 3.63, *F*(1, 678) = 22.45, *p* < 0.001).

Results of demographic information showed that the two groups did not differ for age, sex and laterality quotient (*p*s ≥ .267), but non-believers revealed higher scores in depression (13.07 ± 8.49) and in anxiety (48.64 ± 11.10) compared with believers (depression: 10.78 ± 8.28, *F*(1, 678) = 12.61, *p* < 0.001; anxiety: 45.75 ± 10.65, *F*(1, 678) = 12.01, *p* < 0.001). Concerning hallucinatory propensity, the same ANOVA was carried out on the single scores used to obtain the auditory hallucinations index (i.e. CAPS, AHRS, HE, O-LIFE, HPSVQ). Results were significant only for the AHRS, revealing higher hallucinatory propensity in non-believers (8.16 ± 7.03) compared to believers (6.93 ± 6.41, *F*(1, 678) = 5.75, *p* = 0.017).

## Discussion

This study was first aimed at investigating the phenomenology of auditory hallucinations in an adult Italian sample without neurological and/or psychiatric diagnosis. Second, we aimed to shed light on the possible relationship between non-clinical auditory hallucinations and religiosity. An online survey was created to collect both demographic and psychological measures in addition to standardised questionnaires concerning propensity to experience both auditory hallucinations and religious beliefs. From a demographic viewpoint, we analysed data from 680 healthy adults (age: 18–40), including 28% male responders and 9% left-handers. The vast majority were students (more than 90%) with high school education level (95%), declaring their marital status as single (52%), and about half of them declared a religious belief, with 94% of the believers being Christians. Psychological questionnaires confirmed that on average the sample was free of depressive symptoms (mean score <12 in the BDI-II, in which a score below 13 corresponds to the absence of clinical depression) but revealed a moderate level of trait anxiety (mean score 47 in a range from 1 to 80 in the STAI-Y2).

A qualitative evaluation of the proneness to paranormal experiences showed that at the phenomenological level the sample revealed lower scores on the various auditory hallucination scales (perceptual anomalies measured by the CAPS <8 in a 1–32 range; unusual experience measured by the O-LIFE subscale = 5 in a 0–12 range; phenomenology of auditory hallucinations quantified by means of the AHRS <8 on a 0–40 scale; quantitative features of auditory hallucinations as measured by the HPSVQ <8 on a 0–36 scale), with overall satisfactory internal consistency of the measures. However, a deep analysis of single items revealed that, surprisingly, only 7% of participants reported no hallucinations and/or anomalous experiences at all, with most of them reporting at least one unusual experience in their life. Indeed, on average, less than 30% of the sample reported scores of zero (complete absence of auditory hallucinations) in the questionnaires measuring hallucinatory experiences (HPSVQ, AHRS and HE). This evidence is in line with previous studies on healthy samples: for instance, in a non-clinical UK sample, Bell and colleagues^36^ found a mean score of 7.3 on the CAPS (our sample 7.74), and 5.7 on the Unusual Experience subscale of the O-LIFE (our sample 5.02). Similarly, the results found in the item extracted from a semi-structured interview proposed by Cook and colleagues are in line with this study:^17^ the authors found that in a sample of 58 Christians, 26% reported hearing voices only rarely in their life (30% of our sample), 28% hearing voices relatively often (18% in our sample) and 12% more than 11 times (17% more than once a month in our sample). However, in this item 35% of our sample declared that they had never heard voices, but it must be considered that 51% of our participants declared that they were not believers, whereas the sample tested by Cook was entirely composed of Christian voice-hearers. All these measures of proneness to auditory hallucinations (CAPS, AHRS, HE, O-LIFE, HPSVQ) were correlated to each other, and parallel analysis and PCA suggested the retention of a single factor, with a single component explaining 64% of variance. Indeed, these results, together with internal consistency of each scale, revealed that the questionnaires used correctly framed the proneness to hallucinatory experiences and showed the validity of the scales administered online.

The hierarchic model revealed that 11% of the variance was explained by demographic features of the sample. In particular, being younger, a woman and a non-believer were conditions significantly associated with higher auditory hallucinations, whereas the laterality quotient did not impact on auditory hallucinations. The non-significant result on the laterality quotient is in contrast with the idea of an ‘atypical’ brain lateralisation in persons experiencing auditory hallucinations.^21,23^ Starting from the idea that the cerebral substrate of hearing voices is an anomalous hyperactivity of the linguistic areas in the left hemisphere,^42^ it can be hypothesised that stronger right-handers, who would be mostly lateralised also at a hemispheric level, show a higher propensity for auditory hallucinations. However, it has also been shown that left-hemispheric superiority, as indirectly shown by a stronger right-ear advantage for linguistic contents, is stronger for voices expressing positive contents.^22^ In fact, the expected ear advantage was not found in patients suffering from clinical auditory hallucinations,^23^ where the emotional content is known to be more negative in valence than that reported by healthy individuals.^5^ In conclusion, the relationship between laterality and auditory hallucinations needs to be further explored by using more specific measures of laterality, including neuroimaging and electrophysiological measures.

The positive and negative subscales of the RCOPE^40^ frame different aspects of religiosity, with the positive scale measuring benevolence, spiritual connectedness with others and secure relationships with supernatural forces (i.e. spirituality), and the negative one quantifying spiritual tensions and potential fear of supernatural entities (i.e. similar to persecutory ideation). These two subscales must be considered separately, because they investigate different aspects of spirituality and religion, which can be independent of one another. For this reason, they were inserted in two different steps in the hierarchical linear regression model predicting the auditory hallucinations index obtained from all the auditory hallucinations measures exploited here. Results showed that the negative subscale positively predicted proneness to auditory hallucinations: 7% of variance in the auditory hallucinations index was explained by people’s feeling of being punished or disconnected from God. The sense of connection and intimacy with God (positive subscale) was also positively related with hearing voices or experiencing anomalous perceptions, explaining 1% of variance of the auditory hallucinations index. This result confirms that the relationship between religiosity and unusual experiences is complex: on the one hand, results confirmed higher scores in both negative and positive aspects of religious belief (RCOPE) in believers compared to non-believers; on the other hand, the regression model revealed that religious faith would act as a protective factor for auditory hallucinations. Further studies are needed to disentangle the specific content of auditory hallucinations: it can be hypothesised that negative and positive aspects of religiosity explain different kinds of auditory hallucinations, with the negative component of religious belief possibly related to persecutory contents of voices, and positive component possibly related to positive messages. Since the specific content of auditory hallucinations was not specifically investigated here, this speculation must be explored with questionnaires in further studies.

The final important result of the present study is the crucial role of depression and anxiety in proneness to experience paranormal events. Results of previous studies already suggested a possible link between psychiatric symptoms and auditory hallucinations, but it must be noted that only participants without a psychiatric diagnosis took part in this study. Nevertheless, results revealed that higher scores in the questionnaires measuring anxiety and depression (STAI-Y2 and BDI-II, respectively) explained greater auditory hallucinations scores. This evidence is not surprising and confirms that even in the absence of a specific diagnosis mood alterations are crucial in determining voice-hearing, confirming previous evidence. For instance, Smith and colleagues^33^ found that individuals with stronger depressive symptoms and lower self-esteem had auditory hallucinations of greater severity and more intensely negative content, and were more distressed by them. Delespaul and colleagues^31^ found that anxiety was the most prominent emotion during hallucinations and, importantly, that patients reported high anxiety levels before the first report of auditory hallucinations. We must underline that, differently from the majority of studies on auditory hallucinations, our sample was free from psychiatric and/or neurological diagnosis, but the present results confirm that higher scores in depression and anxiety are significant predictors of auditory hallucinations. This result is even more crucial, considering that when splitting the sample according to the religious belief we found that non-believers showed higher levels of both depression and anxiety compared to believers. This evidence suggests that believing in supernatural forces can exert a protective effect on mood alteration. This result is in agreement with a recent meta-analysis showing, on the one hand, a trend towards association of negative religious coping and greater depressive symptoms and, on the other hand, higher spiritual well-being and protection against depression.^43^ Similarly, a protective impact of religious belief on generalised anxiety disorder had been found.^44^

The direct comparison between the subgroup of believers and that of non-believers also confirmed that being a believer leads to significantly higher scores in the religiosity measures (RCOPE) but, importantly, to lower scores in proneness to auditory hallucinations as measured by the AHRS – even if on the other scales used to assess auditory hallucinations the comparison did not reach significance. AHRS^38^ investigates different dimensions of hearing voices, including loudness, vividness and distress level. Starting from the evidence according to which auditory hallucinations in believers are often experienced as positive and considered as a gift,^12^ we can speculate that this difference is due to the fact that even if the two subgroups do not differ in the frequency of auditory hallucinations (no significant difference emerged, for instance, in the HE assessing the frequency of auditory hallucinations), they differ for the subjective impact of hearing voices, with non-believers being more concerned/stressed by this kind of experience. This speculation could also explain the lower depression and anxiety scores obtained by believers compared to non-believers: as already shown, in fact, spirituality in general can be considered as a coping strategy which can help to enhance resilience towards adverse events in daily life,^14^ and thus to potentially control mood alteration. In this regard, future studies should also consider the specific religious practices of the participants (e.g. frequency of prayer, church attendance, familial religiosity and/or spirituality), together with the duration of participants’ beliefs, to better understand the possible association between spirituality, mood and auditory hallucinations.

### Limitations

Some limitations of the current study must be acknowledged. First, demographic information suggests caution in generalising the present results, due to a possible sample bias (e.g. 90% being Italian students, the majority being females). Subsequent studies should try to compensate for this issue, possibly by means of *ad hoc* recruitment instead of online administration.

Furthermore, as previously mentioned, the specific content of voices was not investigated in this study, leaving open the possibility that specific auditory hallucinations may be differently associated with religiosity and mental health. Future studies should directly explore this possibility to expand our understanding of auditory hallucinations in the healthy population, paving the way for more nuanced interpretations of auditory hallucinations and their broader implications for human cognition.

We adopted the well-established dichotomy of believer vs non-believer, but this categorisation may overlook individuals with broader or less conventional spiritual practices. Also, the majority of our believers reported belonging to Christianity. Future research should include diverse cultural and spiritual perspectives to better understand how these factors influence the phenomenology and meaning attributed to auditory hallucinations.

In conclusion, notwithstanding these caveats, this study makes a significant contribution to our understanding of the phenomenology of auditory hallucinations in healthy adults. We found that personal characteristics, such as age, sex and being a believer, significantly predict auditory hallucinations, whereas the laterality score – as measured by a standardised questionnaire – does not impact on this variable. Moreover, both the negative and the positive component of religiosity significantly impacts on auditory hallucinations, as well as depression and anxiety, which explain a relatively high proportion of the variance in the model, suggesting that alteration in mood, together with persecutory ideas (negative religiosity), could be directly involved in proneness to auditory hallucinations in the healthy population. Further studies are needed in this domain because hallucination-like experiences in the healthy population have received scarce attention so far, but they could become crucial in everyday life. In this view, retrospective studies in clinical populations would be useful to assess whether auditory hallucinations proneness in participants without a psychiatric diagnosis can be a precursor to clinical conditions. Similarly, electrophysiological investigations should be conducted to explore the cerebral basis of non-clinical auditory hallucinations, to verify to what extent the ‘atypical’ brain activity widely shown for clinical auditory hallucinations is present in non-clinical voice-hearers. Moreover, we found that persecutory traits of spiritual beliefs are significant predictors of auditory hallucinations (negative RCOPE explaining a relatively high portion of variance in the model); indeed, this aspect should be further explored to disentangle the direction of the relationship and to investigate whether an attempt to weaken this negative component of supernatural beliefs can mitigate the possible frequency and impact of auditory hallucinations in the non-clinical population. Since the positive aspect of religious belief (positive RCOPE) is also shown to be associated with higher propensity to experience auditory hallucinations (although it explains only 1% of the variance in the model), the exact nature and content of voices heard should be disentangled in future studies to assess to what extent religious faith can be considered a coping strategy against paranormal experiences and mood alterations. The present results confirm that hearing voices is not inherently a psychiatric symptom. However, they also suggest that particular attention should be paid to specific segments of the population: while hearing voices could be considered as ‘a gift’, being a young female with high levels of depression and/or anxiety could form a basis for a negative impact of voices, especially among non-believers. This evidence could be related to the higher proneness found in young female participants (compared to male ones) to experience higher depression and/or anxiety symptoms.^45^ It is also worth noting that a protective effect of religiosity against depression has been suggested only in women.^46^ Similarly, more data about the specific content of auditory hallucinations should be collected to investigate the possible link between positive/negative content of voices and personal distress. All this evidence should be taken into consideration to prevent the onset of psychiatric conditions, designing specific treatments to manage these conditions through psychological and/or psychiatric support.

## Supporting information

Lucafò et al. supplementary material Lucafò et al. supplementary material

## Data Availability

The data supporting the findings of this study are available on request from the corresponding author, I.C.

## Supplementary material

The supplementary material is available online at https://doi.org/10.1192/bjo.2025.10775
