# Supplementary material for: The impact of religiosity, anxiety and depression on proneness to auditory hallucinations in healthy individuals
Source: BJPsych Open. 2025 Jul 22;11(4):e154. doi: 10.1192/bjo.2025.10775 (PMC12303831; doi:10.1192/bjo.2025.10775)
Supplement: Lucafò et al. supplementary material [file S2056472425107758sup001.docx]

**The impact of religiosity, anxiety and depression on proneness to auditory hallucinations in healthy individuals**

**Supplementary materials**

***Descriptive statistics***

*Laterality quotient (Edinburgh Handedness Inventory: EHI)*

The Edinburgh Handedness Inventory (EHI; 1) is a self-report scale used to assess the direction and consistency of laterality preference. We used the 21-item Italian version of EHI (2): participants had to indicate which side of the body they would use to perform a task, e.g. writing, kicking a ball (right, left, both). A laterality quotient is thus obtained, ranging from -100, corresponding to extreme left-handedness, to +100 corresponding to extreme right-handedness (0 corresponding to a complete absence of lateral preference). In the whole sample, the mean laterality quotient was 57.82 (SD: 41.12). Internal consistency for the present research was excellent, Cronbach’s alpha = .940.

*Depression (Beck Depression Inventory: BDI-II)*

The Beck Depression Inventory (BDI) is a self-reporting scale among the most used for measuring the presence and degree of depression (3). We used the Italian version of Beck Depression Inventory®-II (BDI®-II), that contains 21 items on a 4-point Likert scale, from 0 (absence of symptoms) to 3 (severe symptoms). Affective, cognitive, somatic, and vegetative symptoms are included. The minimum score is 0 and the maximum is 63. Scoring is attained by adding the highest ratings for all items, with higher scores denoting stronger symptom severity. Scores 0-13 indicate an absence of depressive contents, between 14-19 indicate mild depression, 27-29 indicate moderate depression and scores 30-63 indicate severe depression. The mean score of the whole sample was 11.91 (SD = 8.45). Internal consistency was satisfactory, Cronbach’s alpha = .876.

*Anxiety (State-Trait Anxiety Inventory: STAI-Y2)*

The State-Trait Anxiety Inventory, STAI-Y2 (4), is a 20-item questionnaire used to measure trait anxiety. We used the Italian version of the questionnaire (5). Using a 1–4 Likert scale, it explores the general feelings of respondents. Ten items are focused on positive feelings and 10 items are focused on negative feelings. Responses on the positive items were reversed, so that higher scores correspond to a higher level of anxiety (range: from 1 to 80). The mean score of the whole sample was 47.17 (SD = 10.96). Internal consistency was excellent, Cronbach’s alpha = .918.

*Perceptual anomalies (Cardiff Anomalous Perceptions Scale: CAPS)*

The Cardiff Anomalous Perceptions Scale (CAPS; (6) is a 32-item self-report scale that measures perceptual anomalies. We used the Italian version of the questionnaire (7). It is designed to assess intrusiveness, distress, and frequency of perceptual distortions. This instrument involves items concerning distortions in perceptual intensity, experiences in all appropriate sensory modalities, and sensory experiences traditionally associated with temporal lobe disturbances. For each item, participants are first asked if they had that experience, answering “yes” or “no”. Only if they answered “yes”, they were further requested to indicate, on a Likert scale from 1 (not at all) to 5 (very much), how much that experience was i) stressful, ii) intrusive, and iii) frequent. For the aim of the present study, we considered only the presence/absence of anomalous experiences, adding the “yes” answers (possible range: 0-32). The mean score of the whole sample was 7.74 (SD = 5.624). Internal consistency was satisfactory, Cronbach’s alpha = .864.

*Unusual Experience subscale (O-LIFE)*

We used the short Italian version of the Unusual Experience scale (8) of the Oxford-Liverpool Inventory of Feelings and Experiences questionnaire (O-LIFE; (9). The subscale includes 12 items about magical thinking, perceptual aberrations, and hallucinations and the final score ranges from 0 to 12, with higher scores suggesting positive symptoms of psychosis. A cut-off is not indicated for the scale, and the mean score of the whole sample was 5.02 (SD = 2.757). Internal consistency was adequate, Cronbach’s alpha = .728.

*Phenomenology of AHs (Auditory Hallucinations Rating Scale: AHRS)*

The Auditory Hallucinations Rating Scale (AHRS; 10) is a questionnaire investigating seven dimensions of AH, measured on a 5- to 9-point Likert-type scale, ranking from lowest (0) to highest intensity (which can be from 5 to 9 depending on the specific item): hallucination frequency (0-9), loudness (0-5), reality/vividness (0-5), number of different voices (0-6), length of hallucinations (from single words to extended discourse, 0-5), attentional salience (how attention-demanding the voice is, 0-5) and distress level (0-5). The final score ranges from 0 to 40, with higher scores corresponding to more severe symptoms. The original questionnaire was translated into Italian and validated with help from a bilingual person. In the validation of the French version of the AHRS (11), carried out with 66 hallucinating patients with schizophrenia, the mean score was 26.02 (SD = 6.08), and the Cronbach's alpha = .61, with a good internal validity. The mean score of our healthy sample was 7.55 (SD = 6.77). Even if a cut-off does not exist for the scale, our sample receives a relatively low mean score, as expected due to the absence of psychiatric diagnosis in the sample. Internal consistency was satisfactory, Cronbach’s alpha = .825.

*Quantitative assessment of AHs (Hamilton Program for Schizophrenia Voices Questionnaire*: *HPSVQ)*

The Hamilton Program for Schizophrenia Voice Questionnaire (HPSVQ; (12) is a 9-item self-report questionnaire developed to yield a quantitative assessment of auditory verbal hallucinations. Respondents indicate how much each item applies to them on a 5-point Likert scale ranging from 0 (least severe or impairing) to 4 (most severe, i.e., causes the greatest amount of disruption and/or disturbance to one's life), and refer to frequency, negative content, loudness, duration, interference with life, distress, impact on self-appraisal, clarity, and compliance with commands. The final score ranges from 0 to 36, with higher scores corresponding to higher proneness of hearing voices. We translated the original questionnaire and validated the translation with help from a bilingual person. In a study with 61 French-speaking voice hearers with psychiatric disorders (13), the authors found a mean score of 24.65 with a Cronbach's alpha = .82. The mean score of the healthy sample we tested was 7.71 (SD = 7.39), and internal consistency was excellent, with Cronbach’s alpha = .934.

*Hallucinatory experience: HE*

To investigate the phenomenon of “hallucinatory experience” (HE) we used a single item from a 13-item questionnaire employed in previous research with spiritual voice-hearers (14). Specifically, the item used was: “*How often do you perceive voices that lack physical reality?*” and the answer was decoded as a 5-point Likert scale: 0: never, 1: rarely, 2: occasionally (less than once a month), 3: often (more than once a month), 4: daily. In the original study (14) Cook found that, in a sample of 58 Christian respondents who reported hearing spiritual voices, 26% reported having heard voices only once in their life; 21% reported 2-4 times; 7% reported 5-10 times; 12% reported more than 11 times (the remaining 35% declared to be uncertain in this regard but they confirmed having experienced AHs at least once during their life). In our sample, 35% of responders declared having never experienced voices (response: 0), 12% hearing voices rarely (response: 1), 18% less than one at month (response: 2), 17% more than one at month (response: 3), and 18% reported to experience AHs daily (response: 4). The mean score of the whole sample was 1.70 (SD = 1.53); internal consistency was adequate with Cronbach’s alpha = .728.

*Religiosity (Positive and Negative Religious Coping: RCOPE)*

The brief Positive and Negative Religious Coping (RCOPE; 15) is a 14-item measure of religious coping with major life stressors, already used in an Italian sample (16). It is divided into two subscales of religious coping, each consisting of 7 items, which identify positive and negative clusters on a 1-to-4 Likert scale, for a final range in each subscale ranging from 7 to 28. The positive cluster reveals a benevolent world view, a sense of spiritual connectedness with others, and a secure relationship with transcendent forces. The negative cluster reflects struggles within oneself, with others, and with the divine, and underlying spiritual tensions. The mean score for the positive subscale (RCOPE-pos) was 11.24 (SD = 5.03), with Cronbach’s alpha = .928; the mean score for the negative subscale (RCOPE-neg) was 10.12 (SD = 3.92), with Cronbach’s alpha =.826.

**References**

1. Oldfield RC. The assessment and analysis of handedness: The Edinburgh inventory. Neuropsychologia. 1971;9(1):97–113.

2. Salmaso D, Longoni AM. Problems in the assessment of hand preference. Cortex J Devoted Study Nerv Syst Behav. 1985;21(4):533–49.

3. Beck AT, Steer RA, Ball R, Ranieri WF. Comparison of Beck Depression Inventories-IA and-II in Psychiatric Outpatients. J Pers Assess. 1996;67(3):588–97.

4. Spielberger CD, Gonzalez-Reigosa F, Martinez-Urrutia A, Natalicio LF, Natalicio DS. The state-trait anxiety inventory. Rev Interam Psicol J Psychol. 1971

5. Pedrabissi L, Santinello M. Verifica della validità dello STAI forma Y di Spielberger. Giunti Organ Spec. 1989.

6. Bell V, Halligan PW, Ellis HD. The Cardiff Anomalous Perceptions Scale (CAPS): a new validated measure of anomalous perceptual experience. Schizophr Bull. 2006;32(2):366–77.

7. Altamura M, Prete G, Elia A, Angelini E, Padalino FA, Bellomo A, et al. Do patients with hallucinations imagine speech right? Neuropsychologia. 2020;146:107567.

8. Prete G, D’Anselmo A, Brancucci A, Tommasi L. Evidence of a Right Ear Advantage in the absence of auditory targets. Sci Rep. 2018;8(1):15569.

9. Mason O, Linney Y, Claridge G. Short scales for measuring schizotypy. Schizophr Res. 2005;78(2–3):293–6.

10. Hoffman RE, Hawkins KA, Gueorguieva R, Boutros NN, Rachid F, Carroll K, et al. Transcranial magnetic stimulation of left temporoparietal cortex and medication-resistant auditory hallucinations. Arch Gen Psychiatry. 2003;60(1):49–56.

11. Dondé C, Haesebaert F, Poulet E, Mondino M, Brunelin J. Validation of the French Version of the Auditory Hallucination Rating Scale in a Sample of Hallucinating Patients with Schizophrenia: Validation de la version française de l’échelle d’évaluation des hallucinations auditives dans un échantillon de patients souffrant de schizophrénie et ayant des hallucinations. Can J Psychiatry. 2020;65(4):237–44.

12. Van Lieshout RJ, Goldberg JO. Quantifying self-reports of auditory verbal hallucinations in persons with psychosis. Can J Behav Sci Can Sci Comport. 2007;39(1):73.

13. Zanello A, Perez AG, Maksimovic J, Wood S, Sentissi O. Validation and clinical usefulness of the Hamilton Program for Schizophrenia Voices Questionnaire (HPSVQ) among French-speaking voice-hearers. L’encephale. 2023.

14. Cook CC, Powell A, Alderson-Day B, Woods A. Hearing spiritually significant voices: A phenomenological survey and taxonomy. Med Humanit. 2022;48(3):273–84.

15. Pargament K, Feuille M, Burdzy D. The Brief RCOPE: Current psychometric status of a short measure of religious coping. Religions. 2011;2(1):51–76.

16. Giaquinto S, Cipolla F, Giachetti I, Onorati D. Italian validation of the Brief Rcope scale for religious coping. J Med Pers. 2011;9(2):70–5.
